# *Streptococcus suis* meningitis following occupational handling of imported frozen pork

**DOI:** 10.1016/j.idcr.2026.e02666

**Published:** 2026-07-07

**Authors:** Hiroo Matsuo, Yui Nakagawa, Mariko Akaogi, Satoshi Kutsuna

**Affiliations:** a Department of Infection Control and Prevention, Graduate School of Medicine, The University of Osaka, Japan; b Department of Clinical Laboratory, Hyogo Prefectural Kobe Children’s Hospital, Japan; c Department of Neurology, Hyogo Prefectural Amagasaki General Medical Center, Japan

**Keywords:** *Streptococcus suis*, Meningitis, Imported frozen pork, Occupational exposure, Infection control

## Abstract

*Streptococcus suis* is a zoonotic pathogen primarily associated with pigs. Although most cases of *S. suis* infection in humans are associated with occupational exposure to live pigs or pork products, as well as consumption of raw pork, infection related to imported frozen pork has been documented in only a few reports. We report the case of a 65-year-old male cook with hand eczema who prepared meatballs daily with his bare hands by using frozen pork imported from Denmark. He developed a fever and headache and showed altered consciousness. Cerebrospinal fluid culture confirmed *S. suis* meningitis. Initial treatment with ceftriaxone, vancomycin, and dexamethasone was subsequently switched to penicillin G based on susceptibility testing results, and the treatment was continued for 14 days. The patient was discharged without complications, such as hearing loss. This case suggests that imported frozen pork following international transport and frozen storage may represent a potential source of *S. suis* infection, underscoring the importance of protective measures such as glove use and strict hand hygiene when handling these products. Thus, clinicians should be aware that zoonotic pathogens may persist in imported frozen meat and pose a risk of causing severe invasive disease.

## Highlights

• *Streptococcus suis* can remain viable for long periods even when frozen.
• Imported frozen pork may be a potential vehicle for *Streptococcus suis* infection.
• Hand eczema increases the risk of *Streptococcus suis* infection in food handlers.
• Protective measures are essential when handling pork during food preparation.

## Introduction

*Streptococcus suis* is a gram-positive coccus, classified into 29 serotypes according to the antigenic structure of its capsular polysaccharide [1]. Initially recognized as a porcine pathogen in 1951 in the Netherlands, it was subsequently reported in the United Kingdom in 1954, and the first case of infection in humans was reported in Denmark in 1968 [2]. To our knowledge, the first case of *S. suis* infection in pigs in Japan was reported in 1979 [3]. In recent years, *S. suis* infection in pigs has been reported worldwide, and cases of infection in humans have been more frequently reported in countries with extensive pig farming [4].

*S. suis* is now recognized as an important zoonotic pathogen, particularly in occupational settings [5]. In pigs, it typically causes meningitis, septicemia, and pneumonia, although asymptomatic carriage is frequent [6], [7]. In humans, the infection most commonly manifests as meningitis or septicemia, with serotype 2 being the predominant cause [6], [8]. Transmission usually occurs through direct contact with pigs or pork products, or by ingestion of raw or undercooked pork [9].

Although most cases are associated with raw (unfrozen) pork exposure, imported frozen pork may also serve as a source of infection. Several reports of *S. suis* infection have been documented in chefs after preparation of pork dishes without adequate protective equipment [10], [11], [12]. Notably, the bacterium can remain viable on pork surfaces during extended transport periods.

Here, we report a case of *S. suis* meningitis in a chef following contact with imported frozen pork.

## Case presentation

A 65-year-old Japanese man with hypertension and a history of peptic ulcer 11 years earlier had been experiencing a fever and chills for 7 days when he visited a local clinic. He had no known conditions associated with impaired immunity, including diabetes mellitus, chronic liver or kidney disease, or malignancy, and he was not receiving immunosuppressive therapy. He reported drinking approximately one bottle of beer daily and had a history of smoking 20 cigarettes per day for 45 years. At this clinic, his illness was treated as a common cold. However, the fever persisted, and on the day of admission, he developed a headache and showed altered consciousness; thus, he was transported to the hospital by ambulance. He was a Chinese cuisine chef who prepared meatballs daily using frozen pork shoulder imported from Denmark. He had longstanding eczema on both palms and handled the meat without any protective equipment. He had no history of pig contact and did not consume raw pork.

## Examination

**Fig. 1:**
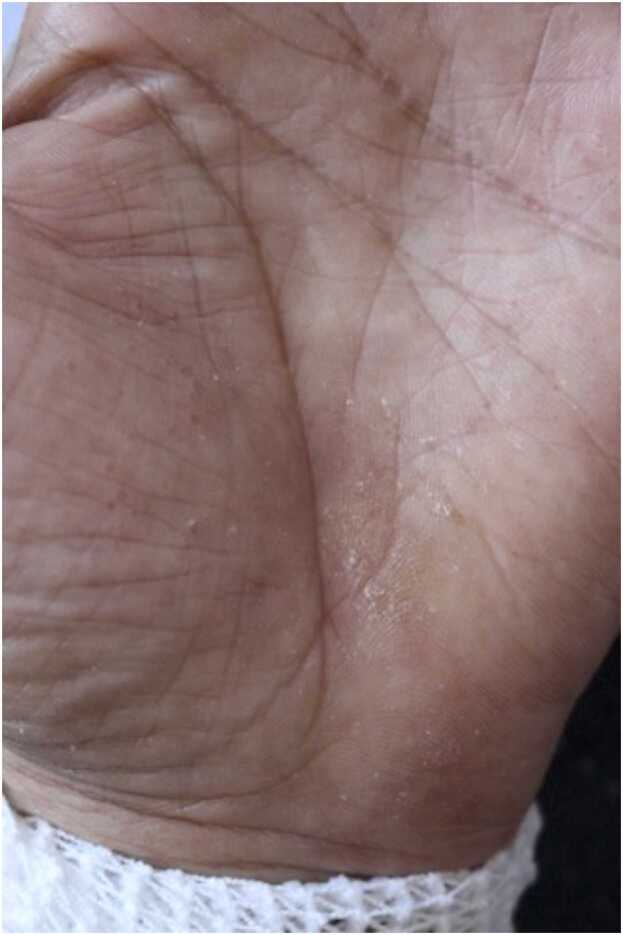
Eczematous lesions on the patient’s left palm, considered as the likely portal of entry for *Streptococcus suis*.

On admission, the patient was febrile (body temperature: 40.2 ℃), with a heart rate of 106 /min, blood pressure of 186/89 mmHg, respiratory rate of 24/min, and oxygen saturation (SpO_2_) of 95% on ambient air. Furthermore, he was disoriented and experienced neck stiffness. Eczematous lesions were evident on both palms (Fig. 1). His laboratory test results showed leukocytosis (white blood cell [WBC] count: 15.6 ×10^9^/L), an elevated C-reactive protein level (12.54 mg/dL), and mildly elevated liver enzymes with preserved renal function. Cerebrospinal fluid (CSF) analysis showed turbidity, elevated opening pressure (21.5 cmH₂O), pleocytosis (WBC count: 137 cells/µL; 99.3% neutrophils), elevated protein (237.7 mg/dL), and decreased glucose levels (26 mg/dL vs blood glucose 139 mg/dL) (Table 1). Brain computed tomography (CT) findings were unremarkable.

**Table 1:** Findings of laboratory and cerebrospinal fluid tests performed on admission.

Test	Result	Reference Range
White blood cells	15.6	3.3–8.6 × 10^9^/L
Neutrophil percentage	95.8	43.0–65.0%
Red blood cells	4.03	4.35–5.55 × 10^12^/L
Hemoglobin	12.8	13.7–16.8 g/dL
Platelets	262	158–348 × 10^9^/L
Total protein	7.4	6.6–8.1 g/dL
Albumin	3.8	4.1–5.1 g/dL
Total bilirubin	0.5	0.4–1.5 mg/dL
Alanine aminotransferase	51	10–42 U/L
Aspartate aminotransferase	39	13–30 U/L
Alkaline phosphatase	204	106–322 U/L
Cholinesterase	222	240–486 U/L
Lactate dehydrogenase	358	124–222 U/L
Gamma-glutamyl transpeptidase	75	13–64 U/L
Amylase	45	44–132 U/L
Creatine kinase	165	59–248 U/L
Blood urea nitrogen	12.0	8–20 mg/dL
Creatinine	0.87	0.65–1.07 mg/dL
Sodium	137	138–145 mmol/L
Potassium	3.8	3.6–4.8 mmol/L
Chloride	101	101–108 mmol/L
Calcium	8.6	8.8–10.1 mg/dL
C-reactive protein	12.54	0.00–0.14 mg/dL
Glucose	139	mg/dL
Prothrombin time	12.4	10.2–13.6 s
Prothrombin	1.11	INR
Activated partial thromboplastin time	26.6	23.0–36.0 s
Fibrinogen	680.0	200–400 mg/dL
CSF opening pressure	21.5	cmH_2_O
CSF white blood cells	137	0–5 cells/µL
CSF neutrophil percentage	99.3	%
CSF total protein	237.7	8–43 mg/dL
CSF glucose	26	mg/dL

## Clinical course

**Fig. 2:**
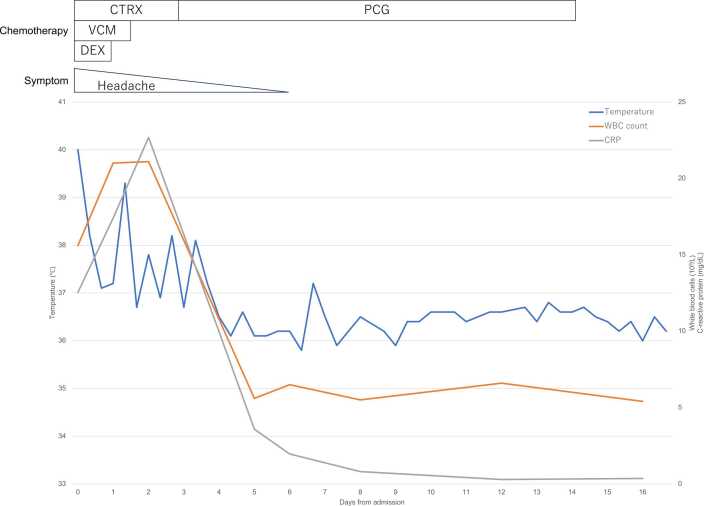
Time course during hospitalization. Temperature, white blood cell (WBC) count, and C-reactive protein (CRP) level are shown in the line graph. The right vertical axis indicates the temperature. The left vertical axis indicates the WBC count and CRP level. The horizontal axis indicates days from admission. CTRX: Ceftriaxone, PCG: Penicillin G, VCM: Vancomycin, DEX: Dexamethasone.

Bacterial meningitis was suspected, and blood and CSF cultures were obtained before initiating empiric therapy with ceftriaxone, vancomycin, and dexamethasone. His consciousness improved the day after the treatment was started. He became afebrile on the fourth day of hospitalization, and his headache resolved by the sixth day. Because his consciousness improved rapidly and because both blood and CSF cultures turned positive within 24 h with rapid testing excluding *Streptococcus pneumoniae*, dexamethasone was discontinued after 1 day of treatment. Treatment was subsequently de-escalated to penicillin G once susceptibility results were available and continued for a total of 14 days (Fig. 2).

Both blood and CSF cultures showed colonies on sheep blood agar, which showed α-hemolysis after 24 h of incubation. The isolate was identified as *S. suis* by using the Sysmex bioMérieux Api 20 Strep system, and identification was further confirmed by *S. suis*-specific polymerase chain reaction [13]. Serum agglutination test and multilocus sequence typing showed that the isolate was serotype 2 and sequence type (ST) 28. Antimicrobial susceptibility testing revealed susceptibility to all β-lactam agents (Table 2). Magnetic resonance imaging performed early during hospitalization revealed no abnormalities, including no inner ear abnormality. Otolaryngological examination also showed no hearing impairment. The patient improved without any neurological complications.

**Table 2:** Antimicrobial susceptibility profile of *Streptococcus suis* isolated from blood and cerebrospinal fluid, showing minimum inhibitory concentrations and interpretations according to the Clinical and Laboratory Standards Institute breakpoints for viridans group streptococci.

Antimicrobial agent	MIC (µg/mL)	Interpretation
Ampicillin	< 0.06	Susceptible
Penicillin	< 0.03	Susceptible
Cefotaxime	< 0.12	Susceptible
Cefepime	< 0.5	Susceptible
Meropenem	< 0.12	Susceptible
Erythromycin	> 2	Resistant
Azithromycin	> 4	Resistant
Clindamycin	> 1	Resistant
Minocycline	4	Intermediate
Levofloxacin	1	Susceptible
Vancomycin	0.5	Susceptible

MIC: minimum inhibitory concentration.

## Discussion

This case emphasizes the clinical significance of imported pork as a potential source of *S. suis* infection. The patient had no contact with pigs and did not consume raw pork; however, he routinely handled imported frozen pork with his bare hands and had eczematous lesions on his palms, suggesting transcutaneous infection during food preparation.

*S. suis* is widely distributed, and asymptomatic carriage among pigs is frequent [7], [14]. Contamination of pork products has been reported in retail markets, with prevalence rates of 6.1% in Hong Kong [15] and 10.8% in Thailand [16]. Similarly, contamination of retail pork with *S. suis* has been reported in Japan [17]. Since 2015, the sale and provision of pork for raw consumption have been prohibited under national food safety regulations in the country. However, most reported human cases in Japan, even before this regulation, have been associated with occupational or handling exposure rather than ingestion [18]. In contrast, in many Southeast Asian countries, raw pork remains available, and consumption of raw pork and pig’s blood has been recognized as a major risk factor for human infection [5], [9]. These observations underscore that in countries where raw pork consumption is limited or prohibited, most cases are linked to occupational exposure; however, imported frozen pork may also represent an underappreciated source of infection, as illustrated by our case.

*S. suis* is capable of surviving for prolonged periods at low temperatures, including several months under frozen conditions [12]. In Shenzhen, China, frozen pork has been reported as a potential source of infection [19]. In the same study, most patients had a history of raw pork exposure, and several cases involved handling raw pork with uncovered hand wounds and without personal protection. In a case–control study conducted in Vietnam, exposure to pigs or pork in the presence of skin injuries was identified as an independent risk factor for *S. suis* infection [9]. In addition, disruption of the epidermal barrier has been associated with an increased risk of systemic bacterial infections, including sepsis [20], [21]. In the present case, the patient had chronic palmar eczema and routinely handled imported frozen pork with bare hands, suggesting that exposure under conditions of impaired skin barrier function may have facilitated the development of *S. suis* infection. However, because microbiological testing of the imported frozen pork was not performed, the association between the pork exposure and the present infection remains circumstantial and cannot be definitively established. Multilocus sequence typing revealed that the isolate belonged to ST28. Whole-genome sequencing studies have demonstrated that ST28 strains circulate in multiple geographic regions, including Japan and Europe [22], [23]. Although ST28 has been reported in both Asia and Europe, and MLST alone does not allow precise determination of geographic origin, this lineage has also been documented in European pigs, including in recent reports from Europe [23]. ST28 strains have demonstrated heterogeneity in virulence in experimental infection models [24]. However, clinical data correlating sequence type with the tempo or severity of human disease remain limited. Although the prodromal phase in this case was relatively prolonged, current evidence does not establish a clear association between ST28 and a more indolent clinical course.

Taken together, the patient’s occupational exposure to imported frozen pork and the absence of other recognized risk factors raise the possibility that handling imported frozen pork contributed to the infection in this case. However, the source of infection cannot be definitively determined.

Nevertheless, because whole-genome sequencing was not performed, a definitive phylogenetic link to a specific geographic source cannot be established.

Such infections have been documented in only a few reports worldwide, highlighting the rarity of this exposure pathway.

This report has several limitations. First, microbiological testing of the pork was not possible because the product had already been consumed. Consequently, direct proof of transmission is lacking. Second, serotype and sequence type data from the pork-producing region were not available, limiting epidemiological correlation. Third, whole-genome sequencing was not performed. Because ST28 circulates in multiple geographic regions, including Japan and Europe, higher-resolution genomic comparison with publicly available strains would have been required to more precisely evaluate transmission links or geographic origin. Therefore, a definitive molecular confirmation of importation was not possible.

## Conclusion

We report a case of *S. suis* meningitis potentially acquired through skin contact with imported frozen pork. This case underscores the need for strict adherence to glove use and hand hygiene when handling pork, including imported products, to prevent zoonotic infection. Clinicians should remain vigilant for this pathogen even in less commonly reported exposure settings, such as handling of imported frozen meat.

## Author agreement

All authors confirm that this manuscript is an original work, has not been published elsewhere, and is not under consideration for publication elsewhere. All authors have read and approved the final manuscript and agree to its submission to *IDcases*.

## Author contributions

Hiroo Matsuo was responsible for the patient’s inpatient care, outpatient follow-up, and drafting of the manuscript. Yui Nakagawa performed the microbiological examinations, including culture, identification, and susceptibility testing. Mariko Akaogi served as the attending physician and was responsible for inpatient management. Satoshi Kutsuna provided critical supervision and guidance in preparing the manuscript.

## CRediT authorship contribution statement

**Yui Nakagawa:** Investigation, Data curation. **Mariko Akaogi:** Investigation. **Satoshi Kutsuna:** Writing – review & editing. **Hiroo Matsuo:** Writing – review & editing, Writing – original draft, Investigation, Conceptualization.

## Informed consent

Written informed consent was obtained from the patient for the publication of this case report.

## Ethics committee approval

Not applicable. Ethical approval was not required according to the institutional review board.

## Funding

This research did not receive any specific grant from funding agencies in the public, commercial, or not-for-profit sectors.

## Declaration of Competing Interest

The authors declare that they have no known competing financial interests or personal relationships that could have appeared to influence the work reported in this paper.
